# Clinical trial design in the era of precision medicine

**DOI:** 10.1186/s13073-022-01102-1

**Published:** 2022-08-31

**Authors:** Elena Fountzilas, Apostolia M. Tsimberidou, Henry Hiep Vo, Razelle Kurzrock

**Affiliations:** 1 Department of Medical Oncology, St. Lukes’s Hospital, Thessaloniki, Greece; 2 European University Cyprus, Limassol, Cyprus; 3 Department of Investigational Cancer Therapeutics, The University of Texas MD Anderson Cancer Center, Houston, TX USA; 4 WIN Consortium, Villejuif, France

**Keywords:** Clinical trials, Precision oncology, Personalized medicine, Real-world data

## Abstract

Recent rapid biotechnological breakthroughs have led to the identification of complex and unique molecular features that drive malignancies. Precision medicine has exploited next-generation sequencing and matched targeted therapy/immunotherapy deployment to successfully transform the outlook for several fatal cancers. Tumor and liquid biopsy genomic profiling and transcriptomic, immunomic, and proteomic interrogation can now all be leveraged to optimize therapy. Multiple new trial designs, including basket and umbrella trials, master platform trials, and N-of-1 patient-centric studies, are beginning to supplant standard phase I, II, and III protocols, allowing for accelerated drug evaluation and approval and molecular-based individualized treatment. Furthermore, real-world data, as well as exploitation of digital apps and structured observational registries, and the utilization of machine learning and/or artificial intelligence, may further accelerate knowledge acquisition. Overall, clinical trials have evolved, shifting from tumor type-centered to gene-directed and histology-agnostic trials, with innovative adaptive designs and personalized combination treatment strategies tailored to individual biomarker profiles. Some, but not all, novel trials now demonstrate that matched therapy correlates with superior outcomes compared to non-matched therapy across tumor types and in specific cancers. To further improve the precision medicine paradigm, the strategy of matching drugs to patients based on molecular features should be implemented earlier in the disease course, and cancers should have comprehensive multi-omic (genomics, transcriptomics, proteomics, immunomic) tumor profiling. To overcome cancer complexity, moving from drug-centric to patient-centric individualized combination therapy is critical. This review focuses on the design, advantages, limitations, and challenges of a spectrum of clinical trial designs in the era of precision oncology.

**Supplementary Information:**

The online version contains supplementary material available at 10.1186/s13073-022-01102-1.

## Background

Traditionally, patients suffering from cancer are treated on the basis of their tumor site of origin and histological subtype. However, rapid advances in molecular technology have yielded clinical-grade tests, unimaginable just a few years ago, that can interrogate each tumor’s omic and immune profile and identify its unique alterations. This information can then be used to provide personalized treatment to patients.

Traditional oncology practice relies on average-population-benefit decisions, often derived from randomized clinical trials of unselected patients, which have been the cornerstone of drug approvals for decades [1, 2]. Customarily, therapy decisions were based on the tumor organ of origin, and this paradigm still often applies. In contrast, precision/personalized oncology depends on data from trials selecting patients on the basis of their genomic/biologic/immune markers. Therefore, some clinical studies are now based on mutation status (e.g., whether the tumor has an *NTRK* fusion or high tumor mutational burden [TMB]) rather than site of origin [3]. Relying on genomics has yielded several tumor-agnostic approvals of agents with remarkable efficacy [3–6].

Both tissue-of-origin studies and tumor-agnostic/genomic-based studies are drug-centered, meaning that the trial offers the same drug(s) to each patient and the patient must fit the trial. Patients are chosen for the trial based on their commonalities, that is, whether they have the same tissue-of-origin diagnosis or, more recently, whether they have the same genomic alteration regardless of tissue of origin. However, as omic technology has improved and become more widely used, a challenging reality has been demonstrated—individual tumors, especially when metastatic, are complex and differ from each other [7, 8]. Due to this significant tumor heterogeneity, optimal therapy requires customization to the individual. Therefore, next-generation trials need to be patient-centered (i.e., therapeutic agents matched to patients based on their tumor biomarkers) rather than drug-centered (i.e., patients matched to specific clinical trials). Traditional randomized, drug-centered clinical trials are important because they attenuate the influence of confounders; however, they are also cumbersome, are costly, and require large numbers of patients to demonstrate clinical benefit [9]. Newer biomarker-based clinical trials have been associated with improved rates of response, progression-free survival (PFS), and overall survival (OS) compared to clinical trials that did not use a biomarker to select patients [10–12].

Patient-centered trials evaluate the robustness of the strategies for allocating N-of-one personalized therapies, rather than evaluating the therapies themselves, since the treatments may differ from patient to patient [13–15]. In addition to novel patient-centered N-of-one trials, advanced computing capabilities, almost inconceivable a few years ago, are yielding altogether new ways to assess drug efficacy. Two such advances are the use of real-world data, which has yielded, in part or in whole, at least two Food and Drug Administration (FDA) cancer drug approvals [16, 17], and the use of registries that collect, store, and analyze large volumes of structured patient data [18, 19]. These registries may enable the evaluation of diverse variables efficiently and at a lower cost. Computer applications also can now record outcomes originating directly from patients, including symptoms, treatment toxicities, and quality-of-life measurements. These data are being digitally collected via automated telephone systems, downloadable applications, or web-based platforms. The massive amount of clinical, molecular, and outcome data often produced in innovative trials requires complex analyses. Artificial intelligence (AI) and machine-learning algorithms are implemented to overcome complexity issues. AI and machine-learning technology can be exploited to capture multi-dimensional data sets, to extract information from various databases, and to analyze and generate statistically valid drug sensitivity prediction models at all stages of drug development [20]. Implementation of AI and machine-learning algorithms can yield a better understanding of the drug mechanism of action and optimize targeted therapy selection, patient enrollment, and stratification (i.e., to identify the right drug for the right patient), thus improving clinical success rates [21–23].

In this review, we describe the design, advantages, limitations, and challenges of clinical trials in precision oncology. We provide examples and results of clinical trials with genomically selected treatments that aim to improve outcomes compared to trials without biomarker-selected treatment. Next-generation clinical trials with superior designs continue to evaluate biomarker-selected targeted treatments as monotherapy and in combination with other agents in selected patients. We also delineate the emerging roles of real-world data, digital applications, structured observational registries, and machine learning and AI in precision medicine. These innovative mechanisms of data collection provide a rich source of clinical, molecular, and outcome data, enabling the association of individual patient and tumor characteristics with clinical benefit from selected treatments.

## Traditional clinical trial design

Traditional phase I cancer clinical trials evaluate the safety and activity of investigational drugs in a relatively small number of patients. Phase II studies examine efficacy (without randomization) and toxicity. Phase III randomized, controlled trials compare the outcome of investigational versus standard therapy. Phase IV trials, also known as post-marketing surveillance trials, assess the safety and efficacy of drugs after regulatory approval. The progress of an investigational drug from phase I to phase III clinical trials is associated with exceedingly high costs, ranging from millions to billions of dollars [24]. Still, the timeframe from starting a phase I trial to receiving FDA marketing approval has decreased in recent years. In 2016, it was reported that this period was approximately 12 years [25], but based on recent FDA approvals, it can be as short as 5 years [25–27]. Furthermore, while drugs have traditionally been approved by the FDA after phase III randomized, controlled trials, in recent years, the FDA has approved drugs earlier, including after only phase I trials in cases where biomarker selection enabled remarkable response rates.

The success rate of randomized clinical oncology trials is ~38% [28, 29]. The gold standard trial for approval is the blinded, randomized study because it minimizes bias. However, randomized trials have their own limitations. For instance, the comparator arm in randomized trials is often suboptimal, at times being comprised of a marginally effective available treatment. As an example, PD-1/PD-L1 inhibitors showed significant clinical benefit in patients with non-small cell lung cancer (NSCLC), but in several trials this was because they were evaluated compared to placebo [30–32]. Additionally, trials assessing promising agents as second-line treatment for patients with advanced NSCLC have used chemotherapy agents with modest clinical benefit, including docetaxel, in the comparator arm (NCT04427072) [33, 34]. Lastly, due to the large variability in patients’ baseline characteristics, comorbidities, and unique tumor molecular profiles and microenvironments, even carefully planned trials are not able to account for all the differences between the randomized arms, resulting in imbalances that may influence outcomes [35]. Therefore, innovative trial designs have been adopted in drug development. These trials address many of the issues above and may be more efficient, but they are not free of shortcomings.

## Precision medicine trials—first generation

### Master protocols

Master protocols include multiple substudies that simultaneously evaluate more than one investigational treatment in patients with cancer and/or a selected treatment in patients with more than one tumor type. Examples include basket, umbrella, and platform trials. Recent data show that the number of master protocols is rapidly increasing [36]. The FDA released a draft guidance for industry with recommendations for conducting master protocol research [37]. Precision oncology trial designs and representative trials are listed in Table 1.

**Table 1:** Precision oncology trial designs and representative trials^a^

Trial design	Representative trials	Design details	Biomarker used	Aim	ORR	Published data (first, last author, reference number)
**First-generation designs**						
**Basket**						
	VE-BASKET	Early phase II	BRAF mutation	Efficacy of vemurafenib in patients with BRAF V600 mutation–positive cancers	NSCLC: ORR 42%, Erdheim–Chester disease or Langerhans’-cell histiocytosis: ORR 43%, colorectal cancer: ORR 0%	Hyman, Baselga [38]
	LOXO-TRK-14001, SCOUT, NAVIGATE	Phase I trials	NTRK fusion	Efficacy and safety of larotrectinib in patients with NTRK fusions	ORR 75%	Drilon, Hyman [5]
	ALKA, STARTRK-1 and STARTRK-2	Phase I-II	NTRK fusion	Efficacy and safety of entrectinib in patients with NTRK fusions	ORR 57%	Doebele, Demetri [6]
	KEYNOTE-016, -164, -012, -028 and -158	Phase II	MSI-H/MMRd	Efficacy of pembrolizumab in previously treated, metastatic MSI-H/MMRd colorectal cancer	All patients combined (*N*=134): ORR 39.6%	Le, André [39] Marabelle, Diaz [4]
	MyPathway	Phase IIa	Alterations in HER2, EGFR, BRAF, and Hedgehog pathway	Efficacy and safety of selected targeted therapies in tumor types that harbor relevant genetic alterations	All patients: ORR 23%, HER2-amplified colorectal treated with trastuzumab and pertuzumab: ORR 38%, NSCLC BRAF V600 treated with vemurafenib: ORR 43%	Hainsworth, Kurzrock [40]
**Umbrella**						
	Lung-MAP (lung)	Phase II, parallel assignment	HRD, c-MET, STIK11, FGFR, Pi3K, RET, KRAS	Efficacy of biomarker-matched target therapies vs “non-match” treatments in patients with advanced lung squamous cell carcinoma	c-MET treated with telisotuzumab vedotin: ORR 9%, Squamous NSCLC treated with durvalumab: ORR 16%, squamous NSCLC homologous recombination repair-deficient treated with talazoparib: ORR 4%	Ferrarotto, Papadimitrakopoulou [41] Redman, Herbst [42] Waqar, Papadimitrakopoulou [43] Borghaei, Papadimitrakopoulou [44] Owonikoko, Gandara [45]
	ALCHEMIST (lung)	Non-randomized, open label, parallel assignment	EGFR, ALK, and PD-L1	Use of genomic profiling in patients with operable lung adenocarcinoma to administer matched therapies and evaluate clonal architecture, clonal evolution, and mechanisms of resistance to therapy	Not applicable (adjuvant)	Govindan, Vokes [46]
	PlasmaMATCH (breast)	Non-randomized, open label, parallel assignment	EDR1, HER2, AKT1, and PTEN	Accuracy of ctDNA testing in patients with advanced breast cancer and ability of ctDNA testing to select patients for mutation-directed therapy	In three different published cohorts ORR varied from 11 to 25%	Turner, Ring [47]
	FOCUS4 (colorectal)	Phase 2–3 randomized	PIK3CA, KRAS, NRAS, TP53, and BRAF	Efficacy of targeted agents in patients with advanced colorectal cancer in molecularly stratified cohorts	Not yet reported	Adams, Maughan [48]
	AML BEAT	Non-randomized, open label, parallel assignment	TET2, IDH1, IDH2, WT1, and TP53	Provides cytogenetic and mutational data to assign patient to a substudy based on the dominant clone	Not yet reported (ongoing)	Burd, Byrd [49]
**Platform**						
	MD Anderson IMPACT1	Navigational	Sequencing and IHC	Use of tumor molecular profiling to optimize the selection of targeted therapies for patients who will participate in a phase I clinical trial program	Patients treated with matched treatment versus not matched: ORR 11% vs. 5%	Tsimberidou, Kurzrock [50] Tsimberidou, Schilsky [51] Tsimberidou, Kurzrock [52]
	TAPUR	Non-randomized, open label	ALK, ROS1, MET, mTOR, TSC, HER2, BRCA, ATM, RET, VEGFR1/2/3, KIT, PDGFRβ, BRAF^b^	Evaluate efficacy of FDA-approved, targeted agents in patients whose tumors have actionable genomic alterations known to be targeted by the respective drug	In three different published cohorts ORR varied from 4 to 29%	Klute, Schilsky [53] Gupta, Schilsky [54] Meiri, Schilsky [55]
	NCI-MATCH	Non-randomized, open label, parallel assignment	EGFR, HER2, MET, ALK, ROS1, BRAF, PIK3CA, FGFR, PTENNF1, cKIT^b^	Evaluate the efficacy of matched targeted treatments in patients with refractory cancers, irrespectively of cancer histology	Patients with HER2 amplification treated with T-DM1: ORR 5.6%, patients with BRCA1/2 mutations treated with wee-1 kinase inhibitor: ORR 3.2%	Azad, Flaherty [56] Jhaveri, Flaherty [57] Kummar, Flaherty [58]
	STAMPEDE	Multi-arm multi-stage, randomized, parallel assignment	No	Evaluate novel approaches for the treatment of men with hormone-naïve prostate cancer	Not yet reported	James, Sydes [59] Parker, Sydes [60] Clarke, James [61]
	MD Anderson IMPACT2^b^	Randomized phase II study	Tumor molecular profiling	Compare progression—free survival in patients with advanced cancer who received matched treatments based on tumor genomic profiling results vs. those whose treatment was not selected based on genomic analysis	Not yet reported (ongoing)	NCT02152254 Tsimberidou, Meric-Bernstam [62]
	I-PREDICT UCSD	Prospective navigation	Molecular alterations, PD-L1, TMB and MSI	Assess whether personalized treatment with combination therapies would improve outcomes in patients with refractory malignancies.	Treatment-refractory, metastatic/advanced with high (>50%) matching score: ORR 45%, first-line, metastatic/advanced [63] and high (>60%) matching score: ORR 40%	Sicklick, Kurzrock [13]
	SHIVA	Randomized, controlled, phase II	Alterations in hormone receptors, and PI3K/AKT/mTOR and RAF/MEK pathways	Assess the efficacy of molecularly targeted treatments matched to tumor molecular alterations versus conventional therapy	Patients with matched vs non-matched treatments: ORR 4.1% vs. 3.4%	Le Tourneau, Paoletti [64]
	NCI-MPACT	Randomized, phase II	Alterations in DNA repair, PI3K and RAS/RAF/MEK pathways	Assess the utility of selecting treatment based on tumor DNA sequencing in patients with advanced cancer compared to not-matched treatment	All cohorts: ORR 2%	Chen, Doroshow [65]
	DART	Multiple cohorts, phase II	Immunotherapy for rare cancers; biomarkers are assessed as correlates	Assess response rates of nivolumab and ipilimumab combination in multiple cohorts of rare and ultra-rare cancers	Four cohorts published: ORR varies from 18% (metaplastic breast) to 44% (high-grade neuroendocrine	Patel, Kurzrock [66] Patel, Kurzrock [67] Adams, Kurzrock [68] Wagner, Kurzrock [69]
**Octopus**	QUILT-3.055	Phase IIb	No	Assess the efficacy of combination immunotherapies in patients who have previously received treatment with PD-1/PD-L1 immune checkpoint inhibitors	N-803 and checkpoint inhibitor: ORR 8% (preliminary data)	Wrangle, Soon-Shiong [70]
**Adaptive**						
	I-SPY 2	Randomized, phase II, parallel assignment	ER, HER2, and MammaPrint	Evaluate multiple concurrent experimental arms and a shared control arm as neoadjuvant treatment of patients with breast cancer using response-adaptive randomization	Not applicable (neoadjuvant)	Barker, Esserman [71] Nanda, Esserman [72] Pusztai, Esserman [73]
	BATTLE-2	Randomized, phase II, single group assignment	KRAS	Identify predictive biomarkers and evaluate the efficacy of matched targeted therapies in patients with non-small cell lung cancer	All cohorts: ORR 3%	Papadimitrakopoulou, Herbst [74]
**Telescope (seamless)**						
	GBM AGILE	Randomized, adaptive, parallel assignment, 2-staged	MGMT	Evaluate multiple agents within patient signatures compared against a common control in patients with glioblastoma	Not yet reported (ongoing)	Alexander, Barker [75]
**Next-generation designs**						
**N-of-1**						
	I-PREDICT UCSD	Prospective navigation	Molecular alterations, PD-L1, TMB and MSI	Assessed the strategy/algorithm used (based on molecular profile) to individualize combination treatments in patients with both refractory and treatment-naïve, advanced lethal cancers	Treatment-refractory, metastatic/advanced with high (>50%) matching score: ORR 45% stable disease>6 months/partial/complete response rate = 50%); First-line, metastatic/advanced and high (>60%) matching score: ORR 40% (stable disease>6 months/partial/complete response rate = 68%)	Sicklick, Kurzrock [13, 63]
	WINTHER	Prospective navigation	Genomics and transcriptomics	Evaluate the use of genomics and transcriptomics to guide therapeutic decisions and individualize cancer treatment	All patients: ORR 11%	Rodon, Kurzrock [15]
	Columbia University Medical Center	Prospective	Whole-genome DNA sequencing and RNA expression analysis	Use tumor profiling to identify actionable molecular alterations possibly targeted by FDA-approved drugs. Treatments are then evaluated on the patient’s tumor tissue, either in cell culture in a patient-derived xenograft)	Not yet reported (ongoing)	Califano [76]
**Home-based trials**						
	ALpha-T	Phase II, single arm, tissue-agnostic	ALK fusion	Evaluate the efficacy and safety of alectinib in patients with ALK-positive advanced solid tumors other than lung cancer	Not yet reported (ongoing)	Kurzrock, Lovely [77]
**Novel mechanisms of data collection**						
**Exceptional responders**						
	Exceptional response to mTOR inhibitor (everolimus)	Translational	Whole-genome sequencing	Investigate the genetic basis of a durable remission of a patient with advanced bladder cancer after treatment with everolimus	Not applicable (selected population with exceptional response)	Iyer, Solit [78]
	Exceptional response to EGFR inhibitor	Translational	EGFR mutation	Evaluate tumor molecular profiling in patients with non-small cell lung cancer with exceptional response to gefitinib to determine underlying mechanisms	Not applicable (selected population with exceptional response)	Lynch, Haber [79]
	Exceptional response to ALK inhibitor	Phase 1 dose escalation trial	ALK fusion	Evaluate safety and efficacy of crizotinib in patients with advanced cancer	All patients: ORR 60.8%	Kwak, Salgia [80]
	Molecular profiling of exceptional responders to cancer therapy	Translational	NA	Identify specific molecular alterations in exceptional responders, unravel mechanisms of response and predictive biomarkers	Not applicable (selected population with exceptional response)	Bilusic, Plimack [81] Wagle, Rosenberg [82]
**Registry protocols**						
	ROOT	Collection of comprehensive data	NA	Create a model of an oncology-centric master observational (registry-type) trial with structured data entry	Not yet reported (ongoing)	Dickson, Kurzrock [18, 19]
**Real-world data**						
	Palbociclib in male breast cancer	Electronic health records	NA	Assess safety of palbociclib in male patients with advanced breast cancer	Not reported	Wedam, Beaver [17]
	Pembrolizumab in part	Electronic health records	MSI/MMR	Assess safety and efficacy in patients with advanced cancer (post-marketing requirement)	Not reported	FDA [83]
	Outcome and toxicity, and economic data for CDK4/6 inhibitors	Prospective-retrospective, and cost analysis	NO	Evaluate clinical outcome, toxicity data and treatment-related costs in patients with advanced breast cancer treated with CDK inhibitors	Not reported	Fountzilas, Koumakis [84]
	Abiraterone acetate plus prednisone for the management of metastatic castration-resistant prostate cancer	Retrospective	NO	Assess treatment failure of patients with metastatic castration-resistant prostate cancer treated with abiraterone acetate plus prednisone	Not reported	Boegemann, Elliott [85]
**Patient-reported outcomes**						
	Measuring Quality of Life in Routine Oncology Practice	Randomized controlled	NA	Assess health-related quality of life, patient satisfaction and patients’ perspectives on continuity and coordination of their care	Not applicable (assess quality of life data)	Velikova, Selby [86]

*Abbreviations*: *ctDNA* circulating tumor DNA, *HER2* human epidermal growth factor receptor-2, *MSI* microsatellite instability, *NA* not applicable, *NSCLC* non-small cell lung cancer, *ORR* objective response rate, *PD-L1* programmed death-ligand 1, *TMB* tumor mutational burden

^a^Note that some trials such as IMPACT, I-PREDICT, and MyPathway fall under more than one category and are therefore listed more than once

^b^Examples of molecular biomarkers used in the trial

#### Tumor-agnostic/gene-specific basket trials

Basket trials are tissue-agnostic trials assessing drugs that target a common pan-cancer gene defect (Additional file 1). More than 30 agents are being evaluated in basket trials [40, 87–89]. Successful examples of gene-directed, histology-agnostic agents include pembrolizumab in mismatch repair deficiency/microsatellite instability-high (dMMR/MSI-H) tumors [4], larotrectinib [5] and entrectinib [6] in tumors harboring *NTRK* fusions, and pembrolizumab for tumor mutational burden-high (TMB-H) tumors [4].

Pembrolizumab was the first agent to receive tumor-agnostic FDA approval, which was based on the results of five single-arm trials (KEYNOTE-016, *n*=58 [90]; KEYNOTE-164, *n*=61 [39]; KEYNOTE-012, *n*=6 [91]; KEYNOTE-028, *n*=5 [92]; and KEYNOTE-158, *n*=19 [4, 93, 94]. Patients with 15 different MSI-H/dMMR tumor types were treated with pembrolizumab. The objective response rate (ORR) was 39.6%, and response duration of ≥6 months was reported in 78% of responders; ORRs were similar between patients with colorectal cancer and other tumor types. FDA approval of the NTRK inhibitor larotrectinib was based on data from three single-arm clinical trials (LOXO-TRK-14001, SCOUT, and NAVIGATE), which enrolled pretreated patients with advanced solid tumors harboring an *NTRK* gene fusion [95]. Among 55 patients (17 tumor types) in the three trials, the ORR was 75% (95% CI 61–85%) [5]. Another NTRK inhibitor, entrectinib, was evaluated in 54 patients (10 tumor types) harboring *NTRK* fusions; the ORR was 57% and the median response duration was 10.4 months [6]. More recently, the FDA granted accelerated approval to pembrolizumab for the treatment of adult and pediatric patients with advanced TMB-H (≥10 mutations/megabase) solid tumors that had progressed on prior treatment [96]. In KEYNOTE-158, a non-randomized, open-label trial, 102 patients with TMB>10 mutations/megabase, pretreated, diverse tumor types received pembrolizumab; ORR was 29%, with 50% of those patients maintaining response ≥24 months [3].

These trials recruited heavily pretreated patients with diverse tumor types, or patients for whom standard treatments had been exhausted. In addition, selected patients who participated in those trials had aggressive cancers, for which treatment options were limited [97–99]. In such patients, later lines of anticancer treatment are expected to yield low response rates [100, 101], but targeted treatments evaluated in the aforementioned trials have been associated with significantly higher response rates than expected from standard treatment.

The significance of genomic biomarkers across tumor types lies in their implementation for selection of an active immunotherapy or gene-directed therapy for many patients whose tumor type would not be individually studied.

An emerging question is whether or not all driver alterations can be successfully targeted across histologies. Co-existing molecular alterations, alternative pathway upregulation, secondary resistance mutations in the original gene, and complex mechanisms of network interactions may lead to intrinsic resistance. For instance, while *BRAF V600* mutations are targetable by BRAF inhibitors in multiple hematologic and solid cancers, ORRs are low in colorectal cancer [102]. It is well established that this limited activity of BRAF inhibitor monotherapy in BRAF-mutant colorectal cancer is associated with activation of epidermal growth factor receptor (EGFR)-mediated signaling [103]. To bypass this resistance mechanism, encorafenib, a potent BRAF inhibitor, was combined with cetuximab, an EGFR antibody, in patients with *BRAF V600E-*mutated advanced colorectal cancer. The use of encorafenib and cetuximab was associated with longer OS (9.3 vs. 5.9 months, hazard ratio (HR)=0.61 [95% confidence interval (CI), 0.48 to 0.77]) and a higher ORR (19.5% vs 1.8%, *p*<0.001) compared to standard chemotherapy [104]. In April 2020, the FDA approved encorafenib in combination with cetuximab for patients with pretreated advanced colorectal cancer harboring a *BRAF V600E* mutation [104, 105]. The improved efficacy of combination therapies may be the result of overcoming tumor resistance when multiple alterations are targeted, including those that evolve as escape mechanisms.

Limitations of tumor-agnostic/gene-specific basket trials include the molecular complexity of defining tumor driver alterations and their interactions that result in resistance to targeted therapies, the lack of a comparator, and the difficulty of accrual across multiple tumors. A particular challenge is the rarity of certain molecular alterations in diverse tumor types.

#### Umbrella trials

Umbrella trials evaluate multiple treatments in different genomic/biomarker subsets for a single histology (Additional file 1). A significant advantage of this design is the concurrent evaluation of the efficacy of multiple distinct treatment regimens in a specific tumor type, thus addressing inter-patient heterogeneity. The umbrella design requires the accurate selection of driver alterations and appropriately matched therapeutic agents that effectively target the alterations. Therefore, the development of multiplex assays with high analytical validity and sensitivity is critical to accurately identify patients for each treatment arm. The selection of treatments matched to each biomarker should be based on robust preclinical data. Umbrella trials can include single or randomized arms. Randomization allows for distinction between the prognostic (reflects outcome of the underlying disease) and predictive (reflects therapy impact on outcome) role of the biomarker used in that treatment arm.

Sometimes, umbrella trials are limited by suboptimal matching of mutations to targeted therapies and the weak effect of selected targeted agents on driver mutations. For instance, in the National Lung Matrix Trial, one of the largest umbrella trials in NSCLC, 2007 of 5467 screened patients were molecularly eligible for the study; 302 patients received genotype-matched therapy [106], but only a few treatment matches provided clinically relevant benefits. For instance, the ORR in patients with MET exon 14 skipping mutation treated with crizotinib was 65% (8 of 12 patients). In subgroups of patients with ROS1 fusions treated with crizotinib, and in those with EGFR T790M mutations treated with osimertinib, the ORRs were 68% and 76%, respectively. Finally, the combination of docetaxel and selumetinib had promising preliminary results in patients with lung adenocarcinoma with NF1 loss; the ORR was 28.6% and the disease control rate was 50% [106]. Importantly, advanced tumors often harbor several molecular alterations and rapidly evolving subclones that limit response to monotherapy. However, for selected targeted therapies that have been successfully matched to actionable molecular alterations, a significant proportion of patients demonstrate at least some regression, and disease control can last for 2–3 years or more in some cases [5, 6, 79, 80], underlying the importance of robust predictive biomarkers.

Umbrella trials are particularly difficult in rare diseases, because molecular subsets might be extremely small and patient recruitment is therefore limited; hence, these trials may take a long time to complete. Moreover, patients with multiple molecular alterations may be eligible for treatment on numerous arms. Finally, the development and validation of a multiplex biomarker assay is more complex than that of a single biomarker.

Successfully conducted umbrella trials include the Lung Cancer Master Protocol (Lung-MAP) [41] and Adjuvant Lung Cancer Enrichment Marker Identification and Sequencing Trial (ALCHEMIST) [46] in patients with lung cancer and I-SPY-2 [107] and plasmaMATCH [47, 108] in patients with breast cancer. Specifically, the Lung-MAP trial aimed to improve enrollment efficiency by using a 200-gene molecular profiling assay to match patients to multiple trial substudies; the protocol was amended to add or remove drugs, depending on drug performance [41]. The trial comprises a screening phase, in which pretreated patients with NSCLC undergo molecular profiling, and multiple biomarker-driven substudies that are conducted and analyzed independently. The Lung-MAP study also included cohorts with non-matched treatments. Results of selected cohorts of patients have been published. The addition of ipilimumab to nivolumab was not associated with improved PFS or OS in patients with advanced NSCLC, not previously treated with immunotherapy [109]. The combination of durvalumab and tremelimumab had minimal activity (ORR, 7%) in patients with advanced NSCLC that had previously progressed on immunotherapy [110]. The use of the poly (ADP-ribose) polymerase (PARP) inhibitor talazoparib in patients with squamous lung cancer with homologous recombination repair deficiency was associated with an ORR of 4% [45]. The Lung-MAP trial exemplifies the performance of large-scale platform studies, based on genomically driven treatments.

Results of I-SPY-2 led to accelerated drug development with seamless transition from phase 1 to phase 3 studies [107]. Additionally, the PlasmaMATCH study demonstrated that blood-derived circulating tumor DNA (ctDNA) analysis can efficiently select patients with advanced breast cancer for mutation-directed therapy. PlasmaMATCH is important because ctDNA analysis offers a non-invasive, cost-effective alternative to tumor biopsy [47, 108] for identifying tumor genomic alterations prior to clinical trial enrollment [111, 112].

#### Platform trials

Master protocols are considered platform trials when they allow the evaluation of multiple hypotheses in a single protocol, yielding faster results at a lower cost. The design of platform trials can be highly variable. For example, certain trials incorporate Bayesian algorithms that permit adaptative decisions, such as expanding or deleting study arms while the trial is running [71]. The Bayesian algorithms, in effect, randomize patients to different arms and select the “best-performing” arm for further follow-up after a handful of patients have been treated. Assessment of the endpoint is continuous, and data are re-analyzed with the addition of each patient. Therefore, as defined by the study design, the efficacy of an interventional agent is determined via interim analyses. Other platform designs simply permit multiple different arms and a variety of biomarkers in a single trial with the objective of separately assessing the efficacy of each intervention.

The disadvantages of platform trials include the difficulty of implementing complicated designs with administrative and logistical complexity. In selected trials that test multiple hypotheses in large numbers of patients, study completion might require long-term follow-up assessments, sometimes increasing the cost of the studies, though these studies still often allow efficient drug development. The complexity of statistical analysis has been a significant challenge, especially in cases of extremely heterogeneous patient groups.

Below, we describe selected innovative platform trials with unique designs. These precision oncology trials have provided promising results by incorporating biomarker-based treatment selection.

##### MD Anderson IMPACT1 and 2

The Initiative for Molecular Profiling in Advanced Cancer Therapy 1 (IMPACT1) trial, started in 2007, was the first genomically driven platform trial in precision oncology across tumor types [50–52, 113]. IMPACT1 demonstrated that patients with refractory cancer could be successfully navigated to matched targeted therapy in clinical trials, with improved outcomes compared with patients enrolled in the same trials without matching [50]. Patients with pretreated advanced cancers who received treatment matched to actionable tumor genomic alterations, compared with patients treated with non-matched therapy, had higher ORR (matched 16.4% vs unmatched 5.4%, *p* < .0001), longer PFS (4.0 vs 2.8 months, *p* < .0001) [47, 108–110], and a higher 10-year OS rate (6% vs. 1%, *p*<0.0001) [113]. Following the encouraging results of the MD Anderson IMPACT1 study, the IMPACT2 trial was initiated in 2014 [114]. IMPACT2 is a randomized clinical trial evaluating the use of tumor molecular profiling to select targeted therapy in pretreated patients with advanced cancer (NCT02152254). The endpoint of the study is to determine whether patients treated with a matched targeted therapy selected on the basis of genomic alteration analysis of the tumor have longer PFS than those whose treatment is not selected on the basis of alteration analysis. A multidisciplinary tumor board recommends potential treatments [62]. In the IMPACT2 trial, in contrast to IMPACT1 and other precision oncology platform trials, patients are randomly assigned to receive treatment selected on the basis of genomic alteration analysis of the tumor versus treatment that is not selected on the basis of alteration analysis. Such a design presents various challenges. In order to overcome barriers due to randomization, the study protocol was amended to include a “patient-preference” cohort for each treatment arm for patients who decline randomization. Importantly, the trial’s adaptive design enables patient recruitment despite evolving tumor biomarkers and the plethora of investigational drugs throughout the years.

##### TAPUR

The Targeted Agent and Profiling Utilization Registry (TAPUR) study, started in 2016, was the first precision medicine clinical trial conducted by the American Society of Clinical Oncology (ASCO). This is a non-randomized, multi-basket clinical trial performed across multiple institutions, which evaluates the efficacy and toxicity of FDA-approved targeted treatments, outside their indications, in pretreated patients with advanced cancer (NCT02693535) [115]. Additionally, real-world data on prescribing practices and on the predictive value of these agents are collected. Thirteen targeted treatments, provided by nine pharmaceutical companies at no cost to patients and selected on the basis of Clinical Laboratory Improvement Amendments (CLIA)-certified tumor molecular profiling data, are being examined. The primary study endpoint of this ongoing trial is ORR or stable disease (SD) ≥ 16 weeks, and the secondary endpoint is survival [116]. Depending on the ORR/SD rate in each treatment arm in the first trial stage, either more patients are enrolled in that treatment arm or the cohort is permanently closed. Results of selected TAPUR arms for specific tumor types have been published [117, 118]. TAPUR enables rapid generation of clinical data on the efficacy and toxicity of diverse treatment agents for specific cohorts based on molecular profiling that may be used to trigger larger studies. TAPUR offers access to targeted therapies currently approved for other indications, and tests hypotheses regarding PFS impact after administration of specific molecularly matched therapy in a variety of tumor types. One major difference between this and other similar trials is that TAPUR allows the use of a variety of CLIA-certified laboratories and sequencing platforms for molecular profiling. This flexibility significantly decreases time to enrollment, which is essential for patients with advanced disease. Finally, one of the important aims of the study is to educate physicians about precision oncology and the value of molecular profiling in selecting matched targeted therapy.

##### NCI-MATCH

The NCI-MATCH trial is a phase II, non-randomized trial launched in 2015 by the US National Cancer Institute (NCI) in collaboration with the Eastern Cooperative Oncology Group–American College of Radiology Imaging Network (ECOG-ACRIN) Cancer Research Group (NCT02465060). The NCI-MATCH trial provides targeted agents at no cost to patients and is performed across multiple institutions. It evaluates treatments that target specific tumor molecular alterations in patients with pretreated advanced malignancies, regardless of the primary tumor type [119]. After enrollment, patient tissue samples are tested for molecular alterations that can be targeted by available treatments. If ≥1 molecular alteration is identified, the patient is assigned the treatment that is the most promising. An interim analysis reported the safety of tumor biopsies, rate of participant enrollment, and tumor profiling success rates [120, 121]. Results from NCI-MATCH trial subprotocols have also been reported [56–58, 122–124]. Trial enrollment is dynamically expanding, with new arms opening for accrual. NCI-MATCH and TAPUR are similar in that they both match molecular abnormalities to targeted therapies across a large national network, providing drugs at no cost to patients. This promotes collaboration between academic and community settings while demonstrating the feasibility of large-scale sequencing practices.

##### STAMPEDE

In 2005, the Systemic Therapy in Advancing or Metastatic Prostate Cancer: Evaluation of Drug Efficacy (STAMPEDE) trial was initiated (NCT00268476) [125]. It is a multi-arm, multi-stage, randomized trial evaluating the benefit from the addition of innovative treatments to androgen deprivation therapy in men with advanced prostate cancer. Each treatment arm is compared against the current standard-of-care treatment. Based on interim analyses of failure-free survival, treatment arms that are insufficiently active are dropped. Since 2006, more than 10,000 patients have enrolled in one of six available research arms [126]. The primary endpoint of the study is the safety and efficacy of novel therapeutic strategies versus standard-of-care treatment in men with high-risk, locally advanced or metastatic prostate cancer [60, 61, 125]. STAMPEDE allowed for the enrollment of thousands of patients in multiple arms that were prospectively adopted or dropped depending on efficacy. One of the major strengths of the trial is the evaluation of widely used approved treatment agents. While the trial enables the concurrent evaluation of multiple treatments, comparisons are made with the control arm, not between innovative treatment agents. Therefore, although selected treatments might be shown to be superior to the control treatment, data on the superiority of a treatment compared to other treatments will not be available.

##### DART

The DART (Dual Anti-CTLA-4 and Anti-PD-1 blockade in Rare Tumors) study (NCT02834013; sponsored by NCI and the SWOG cooperative group) focuses on rare and ultra-rare tumors. The trial is evaluating combination immunotherapy in rare cancers and includes over 50 rare cancer histologic types and a molecular arm accruing patients with PDL1 amplification, which is believed to be a biomarker for immunotherapy response. It has accrued almost 1000 patients and is open at almost 1000 sites across the USA. The DART trial provides the opportunity to assess over 50 cohorts of rare and ultra-rare cancers in a single trial and offers local clinical trial access to patients with uncommon cancers and unmet therapy needs.

##### Summary of platform trials

Platform trials have resulted in the treatment of patients with cancer with genomically matched or immune-targeted agents across tumor types (IMPACT1, IMPACT2, TAPUR, NCI-MATCH, DART [for rare cancers]) or for prostate cancer (STAMPEDE). The efficacy of the precision medicine approach was first demonstrated in IMPACT1. That trial deployed tumor molecular profiling to match patients to innovative treatments, when possible, and demonstrated that patients who received matched treatments had improved PFS, ORR, and OS compared to the patients who did not [50–52, 113]. IMPACT2, a prospective randomized study, is ongoing [62]. The primary aim of the study is to evaluate whether patients who receive targeted therapies matched to molecular genomic alterations have longer PFS compared to those whose treatment is not selected based on tumor profiling [62]. The endpoint analysis will be completed at the end of the study.

Results of selected treatment arms of TAPUR [117, 118], NCI-MATCH [56, 57], and STAMPEDE [60, 61, 125] trials have been reported, while multiple treatment arms are still under evaluation. The ultimate goal of these platform trials is the efficient evaluation of multiple treatment arms in parallel as part of a single protocol, thus lowering the costs and yielding efficacy and toxicity data in a timely manner.

#### Specialized master studies

##### SHIVA

SHIVA was the first randomized precision oncology trial to assess the efficacy of molecularly targeted treatments matched to tumor molecular alterations vs. conventional therapy [64]. The primary endpoint of the trial was PFS. There was no difference in PFS between the two treatment arms (matched treatment, 2.3 months vs 2.0 months in the control arm, HR=0.88, 95% CI 0.65–1.19, *p*=0·41). However, the trial had several limitations [64, 127]. For many matches, there was limited biologic rationale. Selection of targeted treatments was suboptimal. Finally, treatment assignments were performed by the treating physician in the control group, while they followed predefined algorithms in the matched group. The example of the SHIVA trial underscores the importance of accurate treatment “matching” based on robust biological rationale. While the trial randomized patients to either receive personalized treatment or not, approximately 80% of the patients were given single-agent hormonal therapy or PI3K inhibitors, limiting the interpretation of the negative results [128]. The trial remains important because it is the first randomized trial in precision oncology across tumor types.

##### NCI-MPACT

In this study, tumor molecular profiling was used to select treatment for patients with advanced cancers who harbored alterations in one of four signaling pathways [65]. Patients were randomized to receive either a matched treatment (veliparib with temozolomide - DNA repair pathway; adavosertib with carboplatin - DNA repair pathway; everolimus - PI3K pathway; or trametinib - RAS/RAF/MEK pathway) or one of the aforementioned treatment regimens selected from the ones not matched in that patient. The objective response rate in the experimental arm was 2% (95% CI, 0 to 10.9%), suggesting that the respective agents were not effective matches. Regarding the NCI-MPACT trial, the negative results emphasize that predictive biomarkers need to be accurately used to inform treatment selection and patients should not be randomly assigned to treatments that are known to be ineffective or associated with severe toxicity. Additionally, development of more effective agents is critical to improve clinical outcomes. This trial also demonstrated a higher rate of patient withdrawals in the control arm compared to the experimental arm (non-targeted 22% vs. targeted 6%; *p*=.038), suggesting that randomization to non-targeted, ineffective treatments in the era of precision medicine can be challenging.

### Octopus trials

Octopus trials, also referred to as complete phase I/II trials, evaluate combinations of multiple agents with a backbone drug (Additional file 1). For instance, the study of combination immunotherapies in patients who have previously received treatment with immune checkpoint inhibitors (QUILT-3.055) is a phase IIb, multicohort study evaluating different combinations of N-803 (a lab-made fusion protein that induces the proliferation and activity of natural killer and cytotoxic T-cells) with PD-1/PD-L1 immune checkpoint inhibitors (NCT03228667) [129]. Treatment is administered to patients with advanced cancer who have already been treated with immunotherapeutic agents. The primary endpoint of the study is ORR, while secondary endpoints include OS, disease-specific survival, and response duration. Regarding the OCTOPUS trial design, preliminary results of the QUILT 3.055 study demonstrated that the IL15 receptor agonist N-803 combined with various checkpoint inhibitors had promising efficacy in patients with diverse tumor types that had previously progressed on immunotherapy [70]. This study design enables the investigation of various treatment arms simultaneously, thus possibly identifying more than one effective drug combination.

### Adaptive design-based studies

Adaptive design enables the dynamic development of studies by dropping ineffective arms early and increasing patient randomization to more effective treatments while improving biomarker selection based on real-time clinical outcomes. Consequently, these trials require fewer participants and shorter follow-up time than traditional randomized trials. The ability to add new arms while eliminating underperforming arms is a significant advantage. However, there are limitations associated with the early elimination of treatment arms, including the lack of convincing data on safety or other secondary outcomes. Logistical complexity and the demand for timely, high-quality, intense statistical monitoring are challenges often encountered in adaptive trials. Finally, constant need to adapt the design may render the interpretation of the results difficult.

In recent years, adaptive design has been increasingly employed in clinical trials. The Biomarker-Integrated Approaches of Targeted Therapy for Lung Cancer Elimination (BATTLE) trial was a phase II trial where patients with advanced NSCLC were assigned to treatment with the greatest clinical benefit using adaptive randomization based on prospectively assessed biomarkers [130, 131]. This trial established the feasibility of biomarker analysis for treatment selection and set the stage for BATTLE-2 (Biomarker-integrated targeted therapy study in previously treated patients with advanced non-small cell lung cancer) [74]. In this randomized, phase II, open-label study, patients with advanced NSCLC whose cancer had progressed on prior platinum-based chemotherapy were adaptively randomized to one of four arms (erlotinib, erlotinib combined with an AKT inhibitor, MEK combined with an AKT inhibitor, or sorafenib). The overall clinical outcomes of BATTLE-2 were not encouraging; however, the trial proved that adaptive randomization based on molecular profiling is feasible.

Another example of an adaptive trial is the Investigation of Serial Studies to Predict Your Therapeutic Response with Imaging and Molecular Analysis 2 (I-SPY-2) trial, a multicenter, phase II, randomized clinical trial [71]. The trial includes multiple treatment arms that concurrently evaluate the efficacy of standard neoadjuvant chemotherapy in combination with innovative drugs compared to standard treatment alone. Treatment arms that were not found to be efficacious were dropped from the study, while others that have shown clinical benefit are moving forward. Moreover, new investigational arms are being added as knowledge in the field evolves. I-SPY-2 was one of the first trials to evaluate the addition of immunotherapy to neoadjuvant chemotherapy in breast cancer. Pembrolizumab added to the treatment of patients with triple-negative breast cancer resulted in a 3-fold increase in pathologic complete response rates (60% vs. 22%) compared to standard therapy alone [72]. This trial is particularly important because it brings novel therapies to patients early in their disease course.

### Telescope (seamless) design

Telescoped clinical trials seamlessly transition from phase I to phase II and/or phase III clinical trials, thus combining all phases, learning and confirmatory, into a single trial (Additional file 1) [132]. Patients enrolled in the learning and confirmatory stages are included in the final analysis, but patients can also be analyzed separately. This design can shorten the duration of a drug’s development and significantly reduce administrative costs. Other advantages of the seamless strategy include selecting only promising agents to be used in the later trial stages, while dropping the ones that fail early; evaluating combinations with other treatment agents; and focusing on responding subpopulations in the next trial stages [132].

In addition to the complex design, challenges are faced when executing a trial with a seamless design. For instance, during the prolonged period required to complete the study, changes in practice may occur and experimental drugs may gain regulatory approval, therefore making the interpretation of the results difficult. Importantly, taking into consideration data from phase I to incorporate into phase II and then phase III, particularly regarding response and toxicity, is challenging since the trial is designed before phase I and II have occurred.

Glioblastoma Adaptive Global Innovative Learning Environment (GBM AGILE) is a multi-arm, seamless, phase II/III, platform trial [75]. This trial includes two stages; the first stage is a Bayesian adaptively randomized screening stage to identify effective therapies on the basis of impact on OS compared with a common control. This stage also assesses clinical indication and biomarker status to identify the population in which the therapy shows the most promise. Highly effective therapies transition in a seamless manner in the identified population to a second confirmatory stage. The second stage uses fixed randomization to confirm the findings from the first stage to support registration [75]. The significance of the GBM AGILE trial lies in the identification of biomarkers predictive of benefit from innovative agents used in this difficult-to-treat patient population. Importantly, this trial uses a seamless design in order to confirm the clinical benefit of selected treatments in a timely manner and provide patients with poor prognosis additional treatment options. Results of the study are awaited.

In summary, telescoped/seamless design allows transition from phase I to phase II and/or phase III studies, without the time needed for writing new protocols and regulatory approval. This process leads to accelerated drug development and early discontinuation of poorly performing arms with inefficient drugs. The limitations of this approach include the trial complexity.

## Precision medicine trials—next generation

### N-of-1 trials—the I-PREDICT and WINTHER trials

In the context of precision medicine, N-of-1 trials are patient-centered trials that evaluate customized/individualized treatment combinations [13, 15] (Additional file 2). Outcomes are usually compared with historical or real-world outcome data and/or the results of higher and lower degrees of matching. When the degree of matching is assessed, it is the algorithmic strategy used to match patients to drugs that is evaluated, rather than the drug regimens themselves, since the latter differ from patient to patient. These types of trials are novel but becoming critical because it is now recognized that patients with metastatic cancer have complex molecular alterations that differ from patient to patient, necessitating an individualized drug regimen. N-of-1 trials in oncology are distinct from those occasionally used in non-oncologic conditions; the latter involve multiple crossovers that are often randomized/blinded in individual patients.

The addition of omic technologies, enabling the analyses of genomic, transcriptomic, immunomic, proteomic, and metabolomic data, to the oncologist’s armamentarium has revealed several molecular alterations that can be successfully targeted by innovative treatments [15, 50–52]. At the same time, molecular diagnostics have unveiled tumor heterogeneity and complexity, thus underscoring the importance of targeting co-existing tumor drivers and co-activated pathways conferring resistance [133]. Therefore, treating patients whose cancers harbor distinct molecular portfolios based only on single commonalities might be a suboptimal approach. N-of-1 studies have focused on the optimization of treatment selection by addressing molecularly complex and heterogeneous cancers with personalized combinations.

The Investigation of Profile-Related Evidence Determining Individualized Cancer Therapy (I-PREDICT) was the first prospective navigation trial that evaluated personalized treatment with combination therapies in patients with diverse refractory tumor types [13, 14, 134] and more recently in therapy-naïve patients with metastatic lethal cancers [63]. A multidisciplinary molecular tumor board suggested customized, multidrug combinations to target aberrations identified in each patient’s tumor by tumor genomic profiling, ctDNA analysis, programmed death-ligand 1 (PD-L1) expression, hormonal status, and TMB and MSI status assessment. A scoring system was used for each patient to calculate a “matching score” (roughly the number of direct and indirect matches for each patient divided by the number of molecular alterations) [134]. A higher “matching score” was associated with higher disease control, PFS, and OS rates [13]. Ongoing N-of-1 trials are evaluating novel combinations in patients with diverse tumor types [13, 14, 63, 76].

Of interest, the WINTHER trial, an international trial conducted by the WIN consortium, was the first precision medicine study to navigate patients to personalized therapy combinations based on either genomics (Arm A) or transcriptomics (Arm B) [15]. The primary endpoint was based on the Von Hoff model, which compares the PFS of individual patients on the trial (PFS2) with the PFS of the same patient on the treatment administered prior to trial enrollment (PFS1) [135]. Higher vs. lower degrees of matching, with either genomic or RNA-based analysis, was associated with improved PFS [15]. Although the trial did not meet the pre-specified primary endpoint (Arm A: PFS2/PFS1 >1.5 in 50% of patients; Arm B: PFS2/PFS1 >1.5 in 40% of patients), it did demonstrate that genomic and transcriptomic profiling can be used for treatment selection and that patients who were well-matched versus poorly matched to therapy did significantly better.

N-of-1 trials aim to optimize treatment selection, including customized combinations, based on the individual patient and tumor characteristics in order to increase treatment efficacy. Limitations of this trial design include lack of a comparator, heterogeneity of treatments, and complexity of analysis to identify the optimal treatment.

### Home-based trials

In an effort to expand telemedicine technologies, an innovative site-less clinical trial design has emerged [136]. Home-based clinical trials are being conducted by several companies in patients with cancer, among other diseases [137]. These trials enable access to drugs for patients who are unable to travel and participate in traditional site-based clinical trials. Additionally, home-based trials facilitate patient recruitment, enable the inclusion of more representative patient populations, and potentially increase enrollment rates by engaging patients to participate from the comfort of their own homes. Patients are in the care of a wide network of investigators and home-health nurses who ensure the well-being of the patient using digital technologies, but also by examining the patients at their home, instead of requiring them to travel to the hospital. Disadvantages may include suboptimal monitoring of adverse events and response to treatment in a timely manner.

One of the first home-based oncology trials is evaluating the efficacy and safety of alectinib in ultra-rare, locally advanced or metastatic, ALK-positive solid tumors (Alpha-T, NCT04644315) [138]. This is a phase II, single-arm, tissue-agnostic trial design. Patient recruitment is run by Science37 [139]. Investigators may request access for individual patients through the study platform. Patients must be willing to comply with study procedures, which include home-based care and visits by mobile nurses. The goal is to provide equitable access to hard-to-reach patients and increase recruitment, while minimizing infrastructure costs and the need for patients to travel and leave their homes. No results have been reported yet.

Home-based clinical trials should not be confused with “just-in-time” trial activation, where a local investigator can rapidly be approved for a study if they see a potentially eligible patient. When trials investigate a specific patient phenotype or tumor genotype, several months may be required to recruit a patient. To overcome this challenge, sites can be activated “just-in-time” to enroll patients who have already been identified as eligible for participation. In this case, the patient needs to be identified early in the treatment course, which is not always feasible, while site activation processes may still need significant time and resources to be completed, especially at academic centers where trial activation often takes a few months. Therefore, while “just-in-time” trial activation may be a useful solution for patient recruitment in cases of rare tumor/patient characteristics, it cannot replace the benefits of home-based oncology trials, wherein much of the recruitment and monitoring is done remotely in order to treat the patient at home.

## Novel mechanisms of data collection

### Exceptional responders

In randomized trials, average clinical outcomes are reported, and the anecdotal exceptional responses of individual patients are often dismissed. The use of molecular profiling might, however, reveal the mechanistic rationale behind the clinical response in an individual outlier with cancer. An important report on exceptional responders included the case of a patient with metastatic bladder cancer who achieved durable remission after treatment with everolimus. Whole-genome sequencing identified a loss-of-function mutation in *TSC1* (tuberous sclerosis complex), known to regulate the mTOR pathway [78]. Similarly, in a patient with metastatic bladder cancer who participated in a phase I trial of pazopanib and everolimus and had a durable complete response to everolimus, tumor whole-genome sequencing revealed molecular alterations in the *TSC1* and *NF2* (neurofibromatosis type 2) genes, which could be associated with exceptional response to the mTOR inhibitor [82]. Another study using whole-exome sequencing of patients with hepatocellular carcinoma reported that the SLC15A2 genomic variation was associated with exceptional response to an inhibitor of angiogenesis, sorafenib [140]. Analysis of five patients with renal cancer who responded to an mTOR inhibitor revealed alterations in TSC1 and MTOR, possibly associated with exceptional response [141]. Finally, one of the most important applications of exceptional responder interrogation led to the discovery of EGFR mutations as predictive biomarkers of clinical benefit from EGFR tyrosine kinase inhibitors [79]. In particular, the EGFR inhibitor gefitinib, whose FDA approval in unselected lung cancer patients was rescinded because of lack of efficacy in post-marketing studies, was re-approved once trials could be done with *EGFR* mutation-based patient selection [135, 142]. The observation of exceptional responders led to the discovery of *EGFR* mutations as the basis of benefit from EGFR tyrosine kinase inhibitors and the unexpected discovery that ALK was a target for crizotinib [143–148].

Trials conducted to interrogate exceptional responders hold the promise of identifying powerful predictive biomarkers of response. Importantly, the study of exceptional responses can also provide useful insight into the underlying mechanisms of action of targeted therapies. Benefits of this approach include the evaluation of a small number of patients per treatment, reduced costs, and rapid time to results and implementation across treatments and tumor types. The study of exceptional responders is often limited by the lack of uniformity in available biomarker data, the difficulty correlating patient characteristics and clinical outcomes, and the rarity of exceptional responders.

### Registry protocols

Despite the critical information that is provided by randomized and other prospective clinical trials, several issues limit the generalizability of their results in the real-world clinical setting. Randomized trials assess treatment regimens in a highly controlled setting and in selected patients, since participation is often limited due to restrictive eligibility criteria. Additionally, treatments/interventions are often evaluated in complex/highly specialized academic/clinical trial environments, thereby rendering results inapplicable to daily clinical practice. Randomized trials often require large numbers of patients, substantial resources, and considerable time to be completed. Finally, these trials are often subject to “outcome reporting bias” (selective reporting of outcomes) [149]. On the other hand, actual real-world data derived from medical or insurance records may be limited in accuracy by the records themselves. To overcome these limitations, analysis of non-randomized observational data from structured cancer registries is now being utilized [18, 19].

Cancer registry protocols represent a rich source of structured data on cancer incidence, patient demographics, treatment patterns, molecular profiling, and clinical outcomes (Additional file 2), [150, 151]. These data enable the assessment of correlations between different parameters. However, registry protocols require entering high-quality data on large numbers of patients accurately and in a timely manner, which can be particularly challenging.

Survival outcomes from cancer registry data analysis may be discordant with those produced by randomized clinical trials [152]. In a comparative effectiveness study, data from 141 randomized clinical trials were compared to data from patients recorded in the National Cancer Database (cancer registry) matching the eligibility criteria of the respective randomized clinical trials. Cox proportional hazards regression models were used to calculate OS. Concordant results between randomized and non-randomized data were reported in 41% of univariable analyses, 46% of multivariable analyses, and 45% of propensity score models. The discordant results suggest that randomized trials with restrictive eligibility criteria and highly structured evaluations may not translate to equivalent outcomes in real-world settings [152].

#### ROOT

The Master Registry of Oncology Outcomes Associated with Testing and Treatment (ROOT) study is a prospective trial where comprehensive clinical, molecular, treatment, and outcome data of patients with diverse tumor types are collected and registered [18, 19]. The ROOT trial is based on the design of a national Master Observational Trial (MOT) that aims to longitudinally assemble structured real-world data on innovative treatments and tumor biomarkers and use AI and machine learning tools to assess possible associations with clinical outcomes [18]. This national trial is just being started.

A major limitation of registry studies is the complexity of data analysis and the need to ensure the comprehensiveness of the structured data collected. AI and machine-learning algorithms and innovative software tools are required to mine and interpret registry data.

### Real-world data

Real-world data have been increasingly used to assess the efficacy and toxicity of treatment agents in patients with cancer. These data are collected from electronic health records, disease registries, patient-based apps, medical claims, and billing data. The analysis of real-world data using advanced computer processing capabilities provides real-world evidence (Additional file 2). Since data on millions of patients are now captured electronically, there is the enticing possibility of applying AI and machine learning to rapidly discover new therapies.

Real-world data are valuable for the assessment of drug efficacy and safety in patient populations that are often excluded from randomized clinical trials, such as patients with poor performance status, older patients, patients with serious comorbidities, or underserved populations who may not be able to travel to an academic center for a clinical trial. Therefore, real-world data often complement the knowledge from traditional clinical trials and verify the generalizability of their results. This approach allows for the collection of data in parallel, including cancer incidence, patient demographics, treatment patterns, molecular profiles, and clinical outcomes, thus enabling the evaluation of possible correlations. The disadvantages of real-world data assessment include inaccurate reporting in medical records, data discrepancies, difficulty in harmonizing medical records, and subjective assessment of benefit from treatments.

The FDA has initiated the Real-World Evidence Program under the Cures Act to evaluate drug effectiveness, support approval of new indications, and support post-approval study requirements, including dose modification, change in route of administration, or addition of a new patient population [153]. Clinical trials often do not allow specific patient populations to participate, thus limiting data on the use of treatment agents in those populations. However, the respective treatment agents are often administered to these populations in daily clinical practice on the basis of clinical safety and efficacy assumptions.

Real-world data surrounding off-label drug administration can be used for additional approval modifications. For instance, palbociclib was initially approved for the treatment of women with advanced breast cancer, in combination with an endocrine treatment, based on the results of the PALOMA-2 and PALOMA-3 trials, which did not include male patients [154, 155]. In 2019, the FDA expanded the approved indications to include men with advanced breast cancer. This decision was based on real-world data on male patients with breast cancer treated with palbociclib, extracted from electronic health records, insurance claims, and the global safety database, along with data from two phase I clinical trials of palbociclib in men with solid tumors [17]. Another FDA approval partially based on real-world data was the use of pembrolizumab for MSI-H solid tumors [153].

In Europe, the European Medicines Agency and European Commission initiated the Adaptive Pathways program to allow for real-world data to support regulatory submissions for drug approval. If confirmed to accurately reflect the anticipated results of clinical trials, real-world data have the potential to dramatically accelerate drug approval.

### Patient-reported outcome measures

Patient-reported outcome measures (PROMs) are reports originating directly from patients regarding their symptoms, treatment toxicities, or health-related quality of life (Additional file 2). These data are now being digitally collected via automated telephone systems, downloadable applications, or web-based platforms. While PROM questionnaires were originally developed for randomized clinical trials, they are now being increasingly used in clinical practice to detect and monitor patient symptoms and drug adverse events. The use of PROMs in patients with cancer has been correlated with improved clinical outcomes, including symptom control [86, 156], better quality of life [86, 157], decreased emergency department visits/hospitalizations [158], and longer survival [158–161]. Additionally, these data can be used for clinical outcome collection, regulatory decision-making, or research purposes [162, 163]. Further, several cancer-specific applications have been developed to monitor patient symptoms and track PROMs [164–166]. In 2006, the FDA issued a draft to guide the industry on how to use PROMs to support labeling claims. The final document was released in 2009, comprising recommendations on the implementation of PROMs in clinical trials [167].

Despite their value, PROMs are rarely helpful in decision-making for drug approval [168]. Several barriers impede their use in clinical practice, including the increased cost of applications used for PROM collection and technical difficulties encountered by patients in using the respective technology. Patients’ lack of medical knowledge may negatively influence their interpretation of clinical events, possibly leading to under-reporting and underestimating symptom severity.

## Challenges and considerations

Innovative trial designs, including trials evaluating high-throughput profiling data and multiplexed omics, real-world data collection from medical records and insurance claims, and registry and platform trials, often yield massive amounts of complicated data. These so-called “big data” are characterized by velocity, volume, value, variety, and veracity (the “5 Vs”) and require AI and machine-learning algorithms to manage complicated analysis issues [169, 170]. Machine-learning tools are used to increase analysis efficiency, populate data models, define designated patient cohorts to be further explored, and improve statistical analysis by identifying and eliminating potential biases (Table 2). Key challenges and their solutions are presented below.

**Table 2:** Challenges and opportunities by trial design

Trial design	Features	Advantages	Disadvantages	Challenges
**Basket**	One molecular alteration, multiple histologies	-Rare molecular alterations -Test treatment in diverse tumor types in parallel	-Alterations are not driver in every tumor type -Different mechanisms of resistance based on tumor type -Lack of comparative arm	Recruiting rare subsets across multiple disease types
**Umbrella**	One histology, multiple molecular alterations	-Biomarker assessment -Improved enrollment rates when biomarker prevalence is low -Parallel evaluation of multiple treatment agents -Flexibility of dropping failing drugs	-Inadequate sample size -Multiple treatments matching molecular alterations -Suboptimal selection of treatment targets	Intra-patient heterogeneity of molecular findings, making it difficult to categorize patients
**Platform**	Combines umbrella and basket features to create broad-based trial	-Allows the addition or exclusion of new investigational arms during the trial -Enables evaluation of multiple hypotheses in a single protocol -Shortens time -Lowers costs	-Complicated design -Administrative and logistical complexity -Long-term nature -High execution costs	Complexity of statistical analysis and of monitoring of extremely heterogeneous patient groups
**Adaptive**	During the course of the study, the trial is changed as data are collected and analyzed	-Drops ineffective arms early -Modifies patient randomization to more effective treatments -Improves biomarker selection -Requires fewer participants -Requires shorter follow-up time	-Complicated design -Administrative and logistical complexity -Miss important secondary outcome data due to early elimination of treatment arms	Dependent on intense statistical monitoring; constant need to adapt the design may make the interpretation of the results difficult
**Telescope (seamless)**	Seamless transition from phase I to II and sometimes to III	-Combines learning and confirmatory stages -Shortens duration of drug development and approval -Reduces administrative costs -Reduces effort -Focuses on promising treatments to be used in later trial stages -Drops failing treatments early -Focuses on responding subpopulations in later trial stages	**-**Design complexity	Designing all phases of the trial (I, II and III) without taking into consideration preliminary data from phase I trial. The long time period required to complete the study, which may be associated with change in practice and experimental drugs that gain regulatory approval in the interim, therefore making the interpretation of the results challenging
**N-of-1**	Personalized combination therapy; Patient-centric trial where each patient gets a customized therapy. The efficacy of the matching strategy rather than the individual therapies is evaluated	-Based on unique patient characteristics and tumor profile -Addresses molecular complexity and heterogeneity -Customized treatment	-Lack of comparator -Heterogeneity of treatments -Complexity of analysis/statistical algorithms	Difficult fit between individualized therapy and the way clinical oncology is practiced wherein physicians often specialize in specific types of cancer Rarity of patient characteristics Need to analyze the robustness of the strategy (algorithm) for matching, rather than drug combinations, since the latter differ from patient to patient
**Exceptional responders**	In-depth understanding of unusual patients	**-**Biomarker identification -Highlights molecular pathways associated with response to treatments	-Rare cases -Requires validation	Lack of uniformity in available biomarker data and correlation with patient characteristics and clinical outcomes
**Registry protocols**	Structured real-world data	-Collection of data in parallel: cancer incidence, patient demographics, treatment patterns, molecular profiling data, and clinical outcomes -Enables correlations -Lower costs	-Complex analysis	Need to analyze the robustness of the strategy (algorithm) for matching, rather than drug combinations, since the latter differ from patient to patient
**Real-world data**	Data derived from electronic medical records and insurance data, as examples	**-**Collection of data in parallel: cancer incidence, patient demographics, treatment patterns, molecular profiling data, and clinical outcomes -Enables correlations -Lower costs **-**Results are representative of population -Safety data on vulnerable subpopulations **-**Accelerates drug approval	-Inaccurate reporting -Data discrepancies -Subjective assessment of benefit **-**Machine learning may improve information synthesis	Lack of structuring of data Inaccuracies propagated in the medical records, due to cloning of notes and lack of routine secondary checks Difficulty harmonizing records to draw conclusions
**Patient-reported outcomes**	Patients report outcomes, often via digital devices	-Improves symptom control -Improves quality of life -Minimizes emergency department visits/hospitalizations -Improves patient survival -Improves physician-patient communication -Increased access to communication with treating team in case of limited access to hospital (rural areas) -Increased access to communication with treating team during COVID-19 pandemic	-Cost of applications -Difficulty in using the technology -Under-reporting of symptom severity -Underestimated symptom severity	Lack of medical knowledge on the part of patients may influence their interpretation of clinical events
**Home-based trials**	Patients stay at home—the trial comes to them rather than having them travel to the trial	-Increased access to innovative treatments in case of limited access to site-based clinical trials -Increased access to innovative treatments during COVID-19 pandemic -Facilitates patients with difficulties in traveling	-Patient recruitment -Monitoring issues -Patient safety	Difficulty in recruiting patients with rare subsets and challenges in proactively engaging their physicians

Highly selected patients in clinical trials are not representative of the ones encountered in routine clinical practice. Electronic health records can address this challenge by integrating valuable data from diverse sources and thus filling in the gaps. For instance, CancerLinQ, a non-profit subsidiary of ASCO, collects and analyzes data from multiple healthcare systems across the USA [171]. It provides real-world data on patient populations that are representative of those encountered in practice, thus reflecting patient variability while enabling analysis for scientific or other purposes [172–174]. Other computational technologies have developed diagnostic and therapeutic algorithms to analyze data from published literature, physician clinical notes, and scientific guidelines, including guidelines from the National Comprehensive Cancer Network (NCCN) Clinical Practice in Oncology. A characteristic example is IBM’s Watson for Oncology, which combines these data with patient medical records to recommend personalized treatment plans for individual patients [175, 176]. The accuracy of Watson for Oncology and other systems for selecting treatments has not yet been proven.

Completion of a clinical trial requires extensive time, while it may lead to failure and show lack of benefit from the treatment under investigation. Machine-learning and AI technology have the potential to accelerate the process of drug development, reducing failure rates, and increasing the accuracy of predictions [20, 177]. These approaches are being integrated into all stages of drug discovery and decision-making in clinical trials. AI technology can facilitate structure-based drug discovery by predicting the 3D structure of the target protein and drug–protein interactions [178] and may help identify resistance mechanisms. Applications include identification of prognostic biomarkers, target validation, and analysis of digital pathology data [20]. Additionally, machine learning has been included in several drug discovery processes, such as molecular virtual screening and modeling; quantitative structure-activity relationship; toxicity prediction; drug synthesis, interaction, release monitoring, or repositioning; identification of bioactivity, and pharmaceutical manufacturing [177]. Concerns regarding decreased use of manpower to complete these analyses, errors associated with AI learning algorithms, and inefficient use of AI should be addressed before its implementation in clinical practice. As for all trials, important variables such as ethnic pharmacogenomic differences, details of individual patient characteristics, and lifestyle factors such as diet need to be taken into consideration to optimize the accuracy of outcomes generated by AI technology and from real-world data. Following the application of AI in other domains, its use for personalized medicine is expected to accelerate discoveries, while harmonization between discovery and clinical implementation and regulatory approvals should be implemented. Despite the clinical benefit often provided by innovative treatments, a significant proportion of patients will not respond or will eventually recur during treatment. The identification of predictive biomarkers is critical to accurately select patients who will respond to specific therapeutic agents while sparing the rest from an inefficacious treatment and unnecessary toxicity. Additionally, efforts need to focus on overcoming resistance mechanisms, including administering treatment combinations [179, 180] or novel agents targeting the molecular alterations associated with resistance [181].

Until recently, the investigation of molecular biomarkers required evaluation of tumor tissue obtained through a tissue biopsy, and in selected patients repeated biopsies would be required throughout the course of the disease to assess the evolution of tumor molecular profiling and investigate the presence of actionable alterations. However, a biopsy obtained from a specific lesion cannot control for tumor heterogeneity, and in a significant proportion of patients, repeated tumor tissue biopsies involve risks and are rarely an option. Blood-derived ctDNA analysis may overcome the challenges associated with tumor heterogeneity, disease evolution, and resistance to treatment [112], as it enables the acquisition of multiple samples longitudinally without the risks associated with invasive tumor biopsies. Patients with advanced, metastatic cancer (versus early-stage malignancies) typically have a higher ctDNA level owing to the higher total systemic tumor burden [182] and shed DNA in the blood may reflect genomic alterations from multiple metastatic sites, thus addressing tissue heterogeneity. These alterations may indicate tumor evolution, tumor sensitivity, and/or resistance to targeted therapies. Additionally, ctDNA analysis is associated with a shorter turnaround time compared to tumor tissue molecular analysis. Timely ordering of ctDNA analysis when response to ongoing therapy starts to diminish and at the time of disease progression may attenuate treatment selection delays [111]. However, its use is limited owing to, at least partially, suboptimal assessment of molecular alterations in patients with low tumor volume and/or tumors whose cellular DNA is not shed well into the bloodstream.

The implementation of precision oncology trials requires novel administrative needs, complicated designs leading to logistic hurdles, limited access to clinical trial centers, stringent trial exclusion criteria, sample size issues in cases of rare alterations, and reproducibility issues. Disparities in socioeconomic status also influence cancer outcomes and are associated with worse survival [183]. An investigation of clinical outcomes of 41,109 patients who participated in 55 trials demonstrated that socioeconomic deprivation was associated with shorter progression-free, overall, and cancer-specific survival [183]. Next-generation precision oncology trial designs offer several opportunities to overcome these challenges. The application of home-based clinical trials may provide access to innovative treatments for patients with limited ability, either physical or financial, to travel to specialized centers. Registry and real-world data trials can collect data on patients with rare molecular alterations or diseases to assess the efficacy, toxicity, and outcome data of various treatments. Recently, ASCO and Friends of Cancer Research published new recommendations to broaden eligibility criteria in clinical trials to increase access for more patients with cancer [184]. Analysis of electronic health records data (CancerLinQ® Discovery database) demonstrated that, by expanding common eligibility criteria for patients with lung cancer, the number of patients eligible to enroll in clinical trials increased two-fold. These findings seem to be applicable across tumor types [172].

## Conclusions and future perspectives

Patients should be considered for clinical trials when the standard treatment is not efficacious any longer, or has minimal impact on survival, or the patient experiences significant toxicity. Remarkable advances in molecular technology, almost unimaginable a few years ago, now permit deep interrogation of tumor biology and customization of patient therapy, which has enabled a revolution in clinical trial design

Multiple factors should be considered when designing clinical trials in oncology to ensure more favorable outcomes. First, complete molecular profiling is necessary to understand the underlying cancer biology and should include immune, DNA and RNA, proteomic, and/or other biomarkers. Second, therapy should be matched to the biology of the tumor, including combinations of drugs to co-target the multiple drivers that are present in most metastatic cancers. Third, innovative trial design with seamless transition from phase I to phase III trial can accelerate drug development and regulatory approval, while lessening administrative paperwork. Fourth, innovative trial designs, including platform studies, umbrella and basket trials, and octopus and adaptive trials, have the common goal of using novel methods and master protocols to answer multiple questions in a single trial and to offer clinical trial access to large numbers of patients on a single trial. The precise type of study used depends on the investigative goals (Additional files 1 and 2). For instance, basket studies are most suitable for evaluating a molecular marker in a tissue-agnostic cohort, while umbrella studies generally concentrate on a single type of cancer but examine multiple subgroups within that histology. Octopus trials focus on examining multiple drug regimens, often in combination with a single backbone drug.

The most recent innovation in trial design is N-of-1 cancer trials. These trials include multiple patients, but each patient is treated in an individual (N-of-1) manner based on a deep understanding of their tumor biology. The strategy of drug assignment, rather than the drug regimens themselves, is assessed. N-of-1 oncology trials therefore differ fundamentally from other trial designs in that they are patient-centered—the drugs are fitted to the patient—rather than drug-centered—the patient is fitted to the drug trial. (N-of-1 oncology trials are also fundamentally different from trials denoted as “N-of-1” in non-oncology patients, despite the similar name, because the non-oncologic trials involve multiple cross overs in the same patient.)

Access to decentralized (home-based) trials is also new and is an important trial design innovation wherein patients can stay at home and participate in the trial remotely. Such trials are likely to enhance patient well-being and also better replicate real-world conditions. Finally, computerized records and capabilities have paved the way for real-world data, as well as data derived from digital applications or large national registries, which, together with machine learning, may hasten the discovery of new treatments and better approximate real-world conditions. Importantly, in order to ensure accuracy, the source data for real-world data must be available and auditable.

Overall, the new generation of precision medicine trial designs yields a variety of response rates. The highest response rates, reaching 75%, are for clinical trials targeting fusions such as *NTRK* using *NTRK* inhibitors in the tumor-agnostic setting, hence reflecting the driver nature of these fusions, and the fact that molecular alterations can be important, at least in some cases, in a range of cancers, independent of site of origin. N-of-1 trials also yield response rates in the range of 45% in refractory cancers, albeit with high degrees of individualized matching between drugs (often used in combinations) and targets (Table 1); these trials highlight the need to address the fact that metastatic cancers often have complex biologic underpinnings and that each cancer may be unique. Overall, the key biologic insights from these trials include the following: cancer is a disease driven by genomic and immune system alterations, and interrogating and then targeting specifically these alterations, rather than simply classifying cancers only by their site of origin, can produce impressive response rates and regulatory approvals. Even so, many cancers are complex and targeting individual molecular alterations is often not sufficient to influence outcome; hence, combinations of matched therapy in an N-of-1 paradigm are needed. Many challenges, however, remain, including determining which molecular alterations are drivers versus passengers, and how to reconcile the precision medicine patient-centered model with the needs for regulatory authorization of new drugs, that are part of a drug-centered paradigm of drug development, as well as how to efficiently but accurately leverage multiple new types of trial design and the availability of millions of datapoints in real-world data in a way that is verifiable and moves the field forward in a rapid and productive way for patients afflicted with cancer.

## Supplementary Information

**Additional file 1.** Selected first-generation precision medicine clinical trial designs (see also Tables 1 and 2). A basket trial is a tissue-agnostic study assessing one drug targeting a specific molecular alteration or pathway across multiple tumor type. An umbrella trial evaluates different treatments matched to molecular alterations in a single tumor type. Complete phase I trial enables comprehensive evaluation of a specific drug in different combination regimens. An octopus trial consists of multiple arms and investigates different combinations of a drug across multiple tumor types. Telescope trials allow seamless transition from phase I to phase II and/or phase III clinical trials, thus combining all phases, learning and confirmatory, into a single trial.

**Additional file 2.** Selected next-generation precision medicine clinical trial designs (see also Tables 1 and 2). An N-of-1 trial assesses different drugs evaluated in the same patient. N-of-1 trials are patient centered and every patient receives a different drug regimen. The strategy (algorithm) of drug assignment, rather than the drugs themselves, is evaluated. Home-based trials enable access to drugs for patients who are unable to travel and participate in traditional site-based clinical trials. In patient-reported outcome measures, patients directly reports data, often via digital devices, regarding their symptoms, treatment toxicities, or health-related quality of life. Registry trial provides a rich source of structured data including cancer incidence, patient demographics, treatment patterns, molecular profiling, and clinical outcomes. Real-world data enables collection of data in parallel and the assessment of efficacy and toxicity of treatment agents.

## Data Availability

Not applicable.

## Abbreviations

ASCO: American Society of Clinical Oncology
ctDNA: Circulating tumor DNA
ECOG-ACRIN: Eastern Cooperative Oncology Group–American College of Radiology Imaging Network
EGFR: Epidermal growth factor receptor
FDA: Food and Drug Administration
EHR: Electronic health records
IBM: International Business Machines
MAM: Multi-arm multi-stage
MSI: Microsatellite instability
NCCN: National Comprehensive Cancer Network
NCI: National Cancer Institute
NF2: Neurofibromatosis type 2
NSCLC: Non-small cell lung cancer
ORR: Objective response rate
PD-L1: Programmed death-ligand 1
PROMs: Patient-reported outcome measures
SD: Stable disease
TMB: Tumor mutational burden
TMB-H: Tumor mutational burden-high
TSC1: Tuberous sclerosis complex
VEGF: Vascular endothelial growth factor
