# Supplementary figures and images for: Clinical trial design in the era of precision medicine
Source: Genome Med. 2022 Aug 31;14:101. doi: 10.1186/s13073-022-01102-1 (PMC9428375; doi:10.1186/s13073-022-01102-1)

## BASKET TRIAL

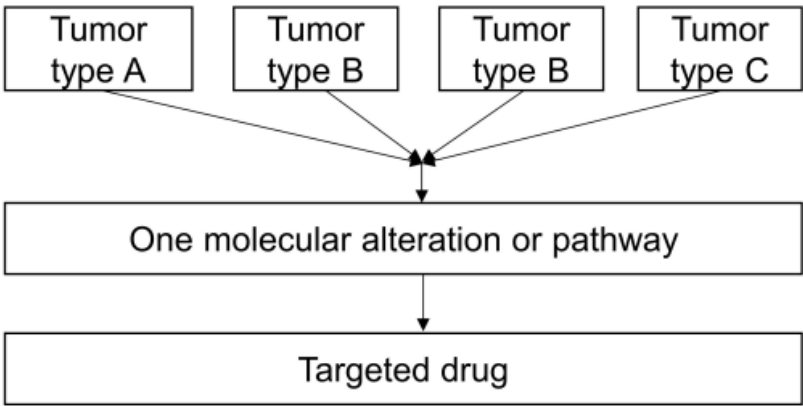

## UMBRELLA TRIAL

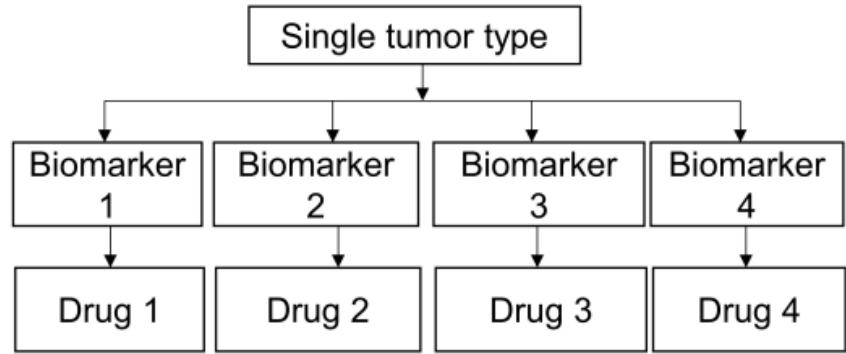

## COMPLETE PHASE I TRIAL

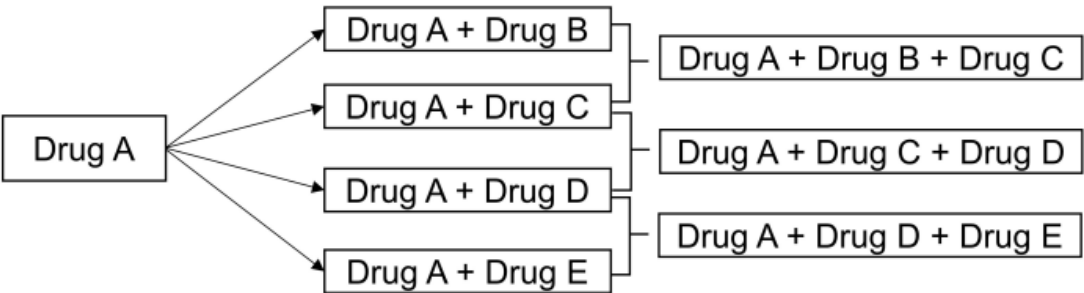

## OCTOPUS TRIAL

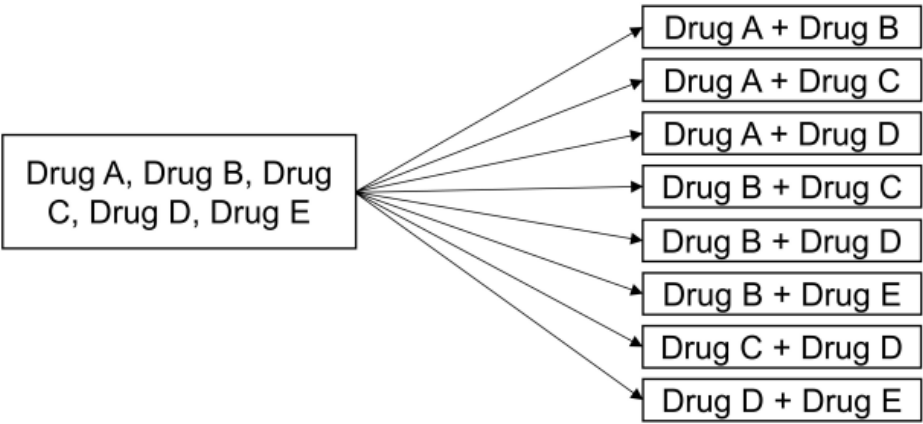

## TELESCOPE (SEAMLESS) TRIAL

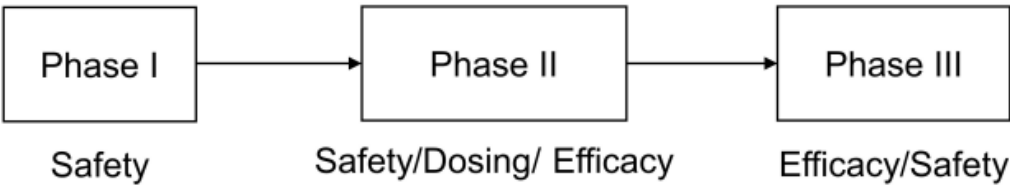

Supplement: Supplementary file 1 — Additional file 1. Selected first-generation precision medicine clinical trial designs (see also Tables 1 and 2). A basket trial is a tissue-agnostic study assessing one drug targeting a specific molecular alteration or pathway across multiple tumor type. An umbrella trial evaluates different treatments matched to molecular alterations in a single tumor type. Complete phase I trial enables comprehensive evaluation of a specific drug in different combination regimens. An octopus trial consists of multiple arms and investigates different combinations of a drug across multiple tumor types. Telescope trials allow seamless transition from phase I to phase II and/or phase III clinical trials, thus combining all phases, learning and confirmatory, into a single trial. [file 13073_2022_1102_MOESM1_ESM.pdf]

# N-of-1 TRIAL

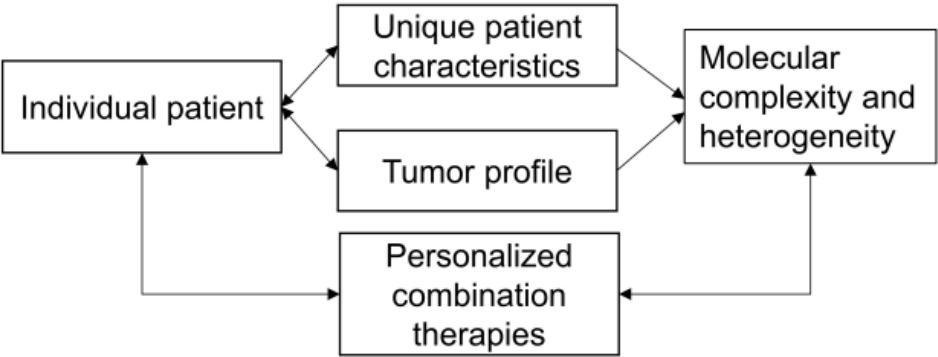

# HOME-BASED TRIAL

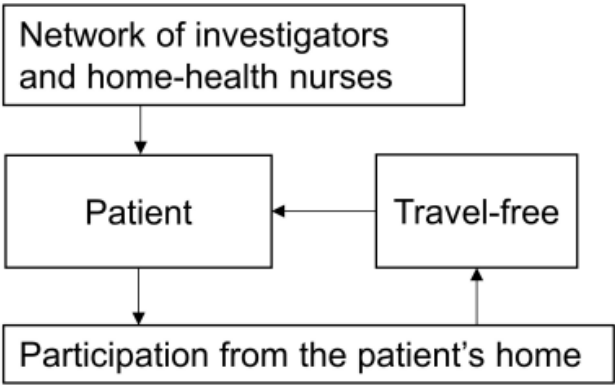

# PATIENT-REPORTED OUTCOME MEASURES

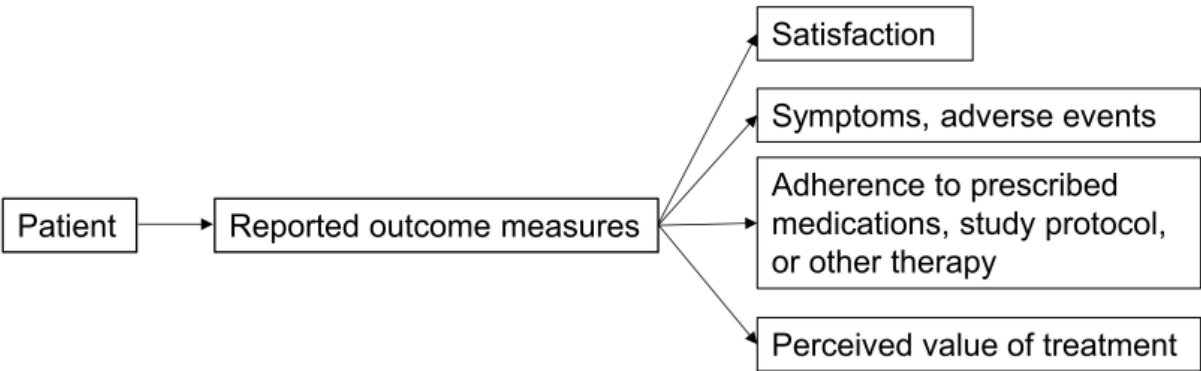

# REGISTRY TRIAL

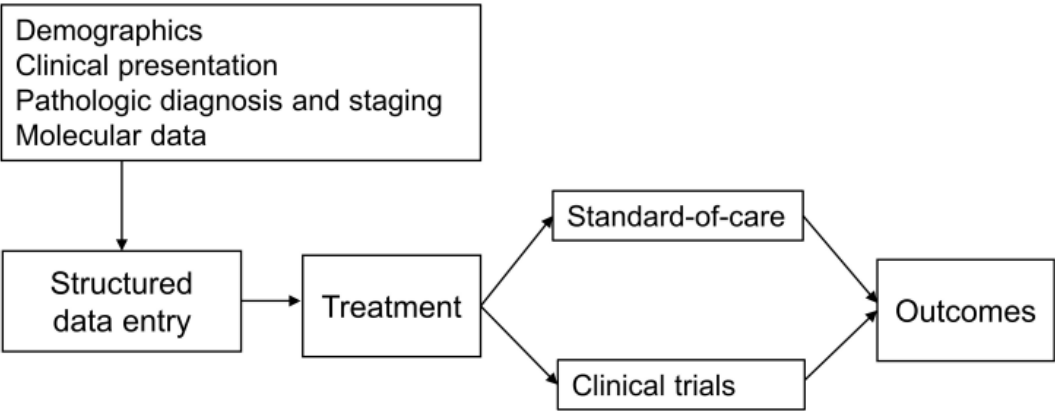

# REAL-WORLD DATA

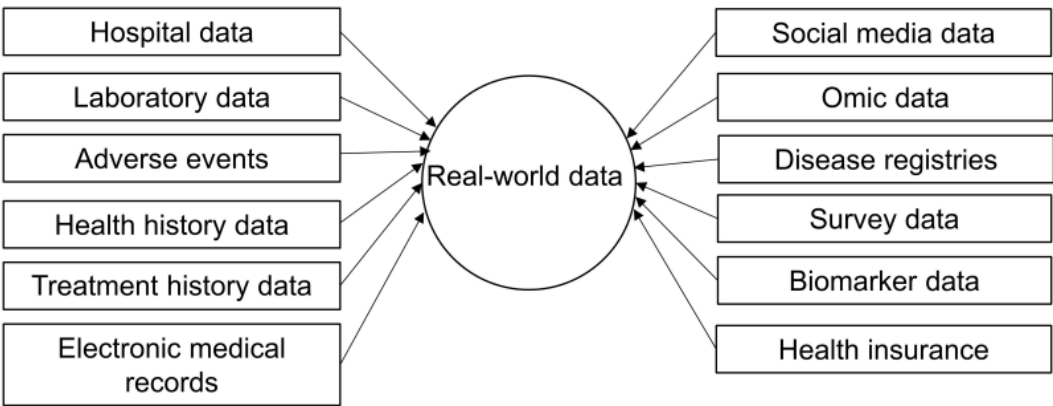

Supplement: Supplementary file 2 — Additional file 2. Selected next-generation precision medicine clinical trial designs (see also Tables 1 and 2). An N-of-1 trial assesses different drugs evaluated in the same patient. N-of-1 trials are patient centered and every patient receives a different drug regimen. The strategy (algorithm) of drug assignment, rather than the drugs themselves, is evaluated. Home-based trials enable access to drugs for patients who are unable to travel and participate in traditional site-based clinical trials. In patient-reported outcome measures, patients directly reports data, often via digital devices, regarding their symptoms, treatment toxicities, or health-related quality of life. Registry trial provides a rich source of structured data including cancer incidence, patient demographics, treatment patterns, molecular profiling, and clinical outcomes. Real-world data enables collection of data in parallel and the assessment of efficacy and toxicity of treatment agents. [file 13073_2022_1102_MOESM2_ESM.pdf]
